# Global Identification of Lunar Dark Mantle Deposits

**DOI:** 10.3390/s26041318

**Published:** 2026-02-18

**Authors:** Xiaoyang Liu, Jianhui Wang, Denggao Qiu, Jianguo Yan, Jean-Pierre Barriot, Yang Luo

**Affiliations:** 1 School of Environmental and Disaster Management, University of Emergency Management, Sanhe 065201, China; liuxiaoyang1209@163.com (X.L.); jianhuiw49@gmail.com (J.W.); 17866926838@163.com (Y.L.); 2 Hebei Key Laboratory of Resource and Environmental Disaster Mechanism and Risk Monitoring, Sanhe 065201, China; 3 State Key Laboratory of Information Engineering in Surveying, Mapping and Remote Sensing, Wuhan University, Wuhan 430079, China; jean-pierre.barriot@upf.pf; 4 Xinjiang Astronomical Observatory, Chinese Academy of Sciences, Urumqi 830011, China; 5 Geodesy Observatory of Tahiti, University of French Polynesia, Faa’a 98702, Tahiti, French Polynesia

**Keywords:** lunar dark mantle deposits, YOLOv8, multi-scale feature extraction, object detection

## Abstract

**Highlights:**

**What are the main findings?**

- A YOLOv8-based deep learning method is proposed for the global identification of lunar dark mantle deposits (DMDs).
- Fifteen newly identified DMD candidates were validated using FeO abundance data and M^3^ spectral absorption features, demonstrating the reliability of the pre-dictions.

**What are the implications of the main findings?**

- Newly identified DMDs account for approximately 9.2% of the global DMD population.
- The updated global distribution shows that 29.8% of DMDs occur in mare regions, while 70.2% are located in the lunar highlands.

**Abstract:**

Lunar dark mantle deposits (DMDs), formed by explosive volcanic activity on the Moon, are typically composed of glass- and iron-rich pyroclastic materials, with slight variations in color, crystallinity, and TiO_2_ concentration by region. This paper proposes a method for identifying DMDs using the YOLOv8 deep learning model, enhanced by the introduction of a multi-scale feature extraction (MSFE) module with an attention mechanism, which improves the model’s ability to detect targets at different scales. First, a DMD dataset was constructed using Lunar Reconnaissance Orbiter (LRO) data, with manual annotations of DMD regions and lunar image slicing to optimize computational efficiency. The YOLOv8 architecture, with the incorporated MSFE module, was then used to improve model accuracy in complex terrain. The experimental results showed that the improved DM-YOLO model achieved a precision (P) of 83.9%, a recall (R) of 83.2%, and a mean average precision (mAP@0.5) of 84.2%, representing increases of 15.2%, 14.4%, and 14.0%, respectively, over those obtained with the original YOLOv8 model. The predicted results were preliminarily verified using FeO abundance data and further confirmed by analysis of M^3^ spectral absorption features, showing strong consistency with known DMDs in terms of both chemical composition and mineralogical characteristics. Observations showed that DMDs were located primarily in the low- and mid-latitude regions of the Moon, with most deposits found in the lunar highlands. The findings suggest that the DM-YOLO model has significant potential for providing technical support for lunar exploration and resource development, particularly for identifying small-scale features that are difficult to annotate.

## 1. Introduction

Lunar dark mantle deposits (DMDs) are important geological features formed by volcanic eruptions on the surface of the Moon, and are primarily composed of black crystalline beads rich in iron and titanium [1]. These deposits are characterized by several factors, including their low albedo, smooth surfaces, and relationship with the underlying topography [2]. DMDs are typically considered products of volcanic explosions or pyroclastic deposits. They mainly formed during early volcanic activity on the Moon when material ejected from volcanic eruptions cooled and settled on the lunar surface, covering some of the younger basalt terrains [3]. Rich in volatile and metallic elements, DMDs are important materials for future lunar resource utilization. Identifying their distribution can provide valuable information for future lunar missions [4]. Furthermore, DMDs are crucial clues for understanding the early internal properties of the Moon. Thus, studying their distribution on the lunar surface helps deepen our understanding of the early volcanic history of the Moon and reveals its tectonic evolution [5].

**Figure 1:**
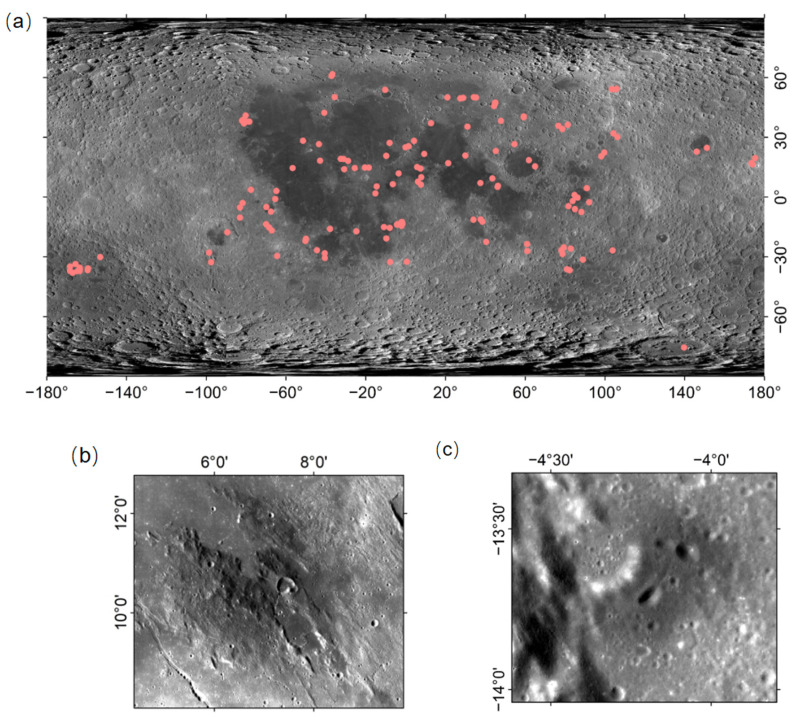
Global distribution and characteristics of dark mantle deposits (DMDs) on the Moon. (**a**) Global distribution map of lunar dark mantle deposits, with data points from [1,2,3,12,13]. (**b**) Regional dark mantle deposits (RDMDs), highlighting the features of these widespread, thin, and flat deposit beds that cover large areas. (**c**) Localized dark mantle deposits (LDMDs), showing the characteristics of smaller deposits that, are typically associated with non-circular volcanic craters or fissures.

Existing studies show the distribution of dark mantle deposits (DMDs) in Figure 1a. In these studies, the deposits are classified into two types: “regional” dark mantle deposits (RDMDs) and “localized” dark mantle deposits (LDMDs) [6]. As shown in Figure 1b, RDMDs are typically thin and flat beds covering an area, and their coverage may reach thousands of square kilometers [7]. In contrast, LDMDs cover smaller areas, usually less than 100 square kilometers, and exhibit distinct positive topographic features [8]. RDMDs typically occur in highlands near continents and are characterized by dark (low albedo) deposits. Their widespread spatial distribution, relationship with the underlying topography, and association with rift valleys and irregular crater-like depressions suggest that these deposits originated from volcanic eruptions [9]. Notably, the smaller LDMDs, as shown in Figure 1c, are often associated with non-circular volcanic craters located at the bottoms of volcanic fissures [10,11]. Dark mantle deposits generally have a dark color and are primarily composed of black crystalline beads rich in iron and titanium, as well as glassy volcanic ejecta [9].

Traditionally, the identification of lunar dark mantle deposits (DMDs) relies on manual analysis of high-resolution remote sensing images. Typically, researchers visually identify dark areas in images and confirm whether they correspond to DMDs by combining spectral and topographic data. However, this method is inefficient, time-consuming, and highly dependent on the expertise of the researcher, making it susceptible to subjective bias. To address this issue, we used the You Only Look Once, version 8 (YOLOv8) model, hoping to provide a new solution for the identification of lunar dark mantle deposits. Currently, many scholars have widely applied deep learning and object detection techniques to identify geological features on the Moon and other planetary bodies. The application of these advanced technologies in planetary science has significantly improved both identification efficiency and accuracy, providing new solutions for the exploration and analysis of the Moon and other celestial bodies. For example, Bickel et al. (2022) constructed and deployed a convolutional neural network to scan approximately 150,000 images from the lunar reconnaissance orbiter (LRO), mapped 28,101 flow features between 60°N and S, and successfully identified lunar particle flows [14]. Tian & Tian (2023), using the YOLOv7 model and integrating the Efficient Squeeze-and-Excitation (ESE) attention module, created a new lunar dome dataset from discrete element method (DEM) data and successfully identified lunar domes [15]. Additionally, Yan (2024) utilized an improved UNet++ and YOLOv5 model combined with remote sensing data to extract linear structures [16]. These studies demonstrate the wide potential of deep learning technologies for identifying geological features on the Moon and other planetary bodies. However, to date, the application of deep learning techniques in detecting lunar DMDs has remained relatively unexplored, highlighting the novelty and significance of the current study.

In this study, we propose a method for identifying lunar DMDs on the basis of the DM-YOLO deep learning model. YOLOv8, with its outstanding detection capability and efficient feature extraction, serves as the core framework for our research. Lunar DMDs present complex geographical features and scale variations, making it challenging for traditional object detection methods to accurately identify these deposits at different scales and with varying terrain conditions. To address this issue, we introduce a multi-scale feature extraction (MSFE) module with an attention mechanism into the YOLOv8 model. The MSFE module is incorporated in a task-driven manner to better accommodate the distinctive morphological characteristics of lunar DMDs, and is designed to extract and fuse feature representations at different receptive field scales. This enables the network to simultaneously capture large-scale morphological context and fine-scale local variations that are critical for DMD identification, particularly under complex geological settings. By integrating an attention mechanism, the module adaptively emphasizes informative features while suppressing irrelevant background responses, thereby improving the representation of diffuse, low-contrast, and scale-variable DMD targets in global lunar imagery. This design significantly improves the identification accuracy of lunar DMDs. After completing the identification task, we further analyze the distribution characteristics of DMDs across the entire lunar surface, providing new insights for subsequent lunar resource exploration and geological studies.

## 2. Data and Methodology

### 2.1. Data Source

In this study, we used multiple datasets, including the Lunar Reconnaissance Orbiter (LRO; National Aeronautics and Space Administration, Washington, DC, USA), Lunar Reconnaissance Orbiter Camera (LROC; Arizona State University, Tempe, AZ, USA), and Wide Angle Camera (WAC) Global Morphology Mosaic, the Clementine UVVIS-derived FeO abundance map (NASA, Washington, DC, USA), the LROC-derived TiO_2_ abundance map (Arizona State University, Tempe, AZ, USA), and Level 2 hyperspectral data from the Moon Mineralogy Mapper (M^3^) onboard the Chandrayaan-1 mission (Indian Space Research Organisation, Bengaluru, India).

The LRO WAC enabled the instrument team to create a global mosaic composed of more than 15,000 images acquired between November 2009 and February 2011 [17]. The LRO WAC Global Morphology Map is a high-resolution lunar surface imagery dataset with a resolution of 100 m, covering the entire surface of the Moon [18]. This dataset, acquired by the LRO WAC, provides detailed lunar morphological information, including features such as craters, maria, and mountain ranges [19]. In our study, this mosaic served as a base layer for manually annotating training samples. Although it suppresses albedo variations to emphasize morphological features, dark mantle deposits (DMDs) remain visually identifiable due to their relatively low reflectance and smooth, crater-poor morphology compared to surrounding terrain. In addition, data volume and processing efficiency are important considerations for global-scale deep learning applications. Higher-resolution datasets such as LROC NAC, SELENE Terrain Camera, and Chang’e optical imagery have substantially higher spatial resolution and data volumes, requiring extensive mosaicking, normalization, and computational resources at the global scale. In contrast, the LROC WAC Global Morphology Map provides a pre-processed and globally consistent mosaic that is well suited for large-area automated analysis and model training.

The Clementine UVVIS-derived FeO abundance map was generated from data collected by the UVVIS imaging spectrometer aboard the Clementine mission and covered a wavelength range from 415 nm to 1000 nm [20]. The derived iron abundance map was created using the algorithm developed by Lucey [21] and later modified by Lawrence [17], with the FeO weight percentage represented by grayscale values ranging from 0 to 25 wt.%. Using specific band ratios and modelling, the FeO abundance on the lunar surface was derived. Although Clementine-derived FeO maps may underestimate iron content in dark mantle deposits, they remain a widely used first-order compositional proxy. In our study, this dataset was used only for qualitative post-prediction validation of model results and was not involved in model training.

The TiO_2_ abundance (weight percentage) was derived from the ratio of the 321/415 nm bands from approximately 124,300 images taken with the LROC WAC between 21 January 2010, and 1 May 2013 [22]. Particularly, these images were combined with the WAC Hapke-normalized mosaic of 321 nm and 415 nm band data to generate a TiO_2_ abundance map [23]. The creation of the TiO_2_ weight percentage abundance map relied on a linear correlation between the TiO_2_ content of lunar soil samples and the WAC 321/415 nm ratio at the sampling sites, converting the 321/415 nm ratio map into a TiO_2_ abundance estimate [24]. While TiO_2_ abundance alone is not a definitive diagnostic indicator of dark mantle deposits, it provides useful geochemical context and was therefore included as a supplementary input to support compositional analysis of the predicted regions.

In addition to these datasets, we used hyperspectral data from the Moon Mineralogy Mapper (M^3^) onboard the Chandrayaan-1 mission for spectral validation. M^3^ is a visible to near-infrared imaging spectrometer that covers a spectral range from 0.4 to 3.0 μm with a spectral resolution of ~20 nm and a spatial resolution of ~140 m/pixel or ~280 m/pixel, depending on the observing mode. For this study, we used the Level 2 Version 1.0 calibrated data archived in the NASA Planetary Data System (PDS) [25]. These data were radiometrically calibrated [26], thermally corrected [27], and photometrically adjusted, especially in long-wavelength regions. M^3^ data were used to examine the reflectance and continuum-removed spectra of selected predicted DMDs, allowing for the identification of diagnostic absorption features near 1000 nm and 2000 nm indicative of Fe-bearing glass or pyroxene.

### 2.2. Data Processing

#### 2.2.1. Building the Dataset

The global morphology map generated by the LROC WAC was obtained, and the coordinates of the lunar DMDs were acquired from relevant literature and websites. Specifically, the initial inventory of known lunar pyroclastic and dark mantle deposits was compiled from previously published geological studies, including Gaddis et al. (1985), Gustafson et al. (2012), Farrand et al. (2023), Besse et al. (2014), and Souchon et al. (2013), which provide spatially referenced locations and geological interpretations of lunar pyroclastic deposits [1,2,3,12,13]. These literature sources served as the primary reference for constructing the initial coordinate database used for manual annotation. These coordinates were subsequently marked in ArcGIS 10.5, as shown in Figure 1a, and the DMD region was cropped using the raster clipping tool to obtain images containing the DMDs. Next, the labelling tool (labelImg) was used to manually annotate the DMD regions in the images The core task of labelling was to outline the DMD regions with rectangular boxes and assign the label “DMD” to each box. To ensure that the labelling file format was correct and that the coordinates were accurate, it was essential to carefully check the consistency between each image file and its corresponding annotation file during the labelling process. Each image had to have a corresponding annotation file to guarantee the integrity of the dataset, which was used for subsequent training of the DM-YOLO model for DMD detection. Each step in the data preparation process was critical. From image collection to the accuracy of labelling, all stages directly affected the performance and detection accuracy of the model.

Finally, the dataset was randomly split into 80% for training and 20% for validation, with strict verification of the annotations to ensure consistency and accuracy, thus providing high-quality data support for model training. The split was performed at the image-tile level after annotation, ensuring that tiles containing DMD features and background regions were proportionally represented in both subsets, while the validation set remained fully independent from the training process. Because DMD regions occupy only a small fraction of the lunar surface compared with background terrain, class imbalance is an inherent challenge in the dataset. To mitigate this effect, the image tiling strategy increases the sampling frequency of regions containing annotated DMDs, and the YOLOv8 training framework employs adaptive loss balancing during optimization to maintain stable learning without introducing manual weighting parameters.

#### 2.2.2. Lunar Image Slicing

To effectively process the global morphology maps captured by the LRO WAC, this study employs image tiling techniques. Although the LROC WAC provides lunar image data with wide coverage and high resolution, the large volume of data can pose challenges in terms of computational resources and memory when performing direct analysis and processing. Therefore, image tiling was used as a preprocessing step to divide large lunar images into smaller segments, improving the efficiency of subsequent analysis and the accuracy of model predictions.

In this study, the image tiling process was implemented in Python using image processing libraries such as OpenCV and Pillow (PIL). First, each image tile was cropped as a square of 610 × 610 pixels, and the global morphology map from the LRO WAC was divided into 63,546 smaller tiles in a grid layout. The tile size was selected to balance computational efficiency, memory constraints, and the input requirements of the YOLOv8 model, while preserving sufficient spatial context for identifying diffuse dark mantle deposits. The size of each tile was adjusted to meet the input requirements of the subsequent model, ensuring that the tiled images matched the input specifications for the YOLOv8 model. During global prediction, adjacent tiles overlap within the mosaic, allowing surface features extending across tile boundaries to be captured and consolidated in post-processing to reduce duplicate detections. Each tiled image segment could then be independently treated as a data sample and input into the deep learning model for prediction. This method not only reduced the demand for computational resources but also allowed for precise analysis of specific lunar surface features or geographic regions, further enhancing the prediction efficiency of the model.

### 2.3. YOLOv8 Model Architecture

The YOLO algorithm used in this study is one of the most widely applied object detection algorithms and has evolved from YOLOv1 to the latest rendition, YOLOv11. The advantage of YOLO lies in its ability to process an entire image within a network, simultaneously detecting multiple targets of various sizes and directly outputting their positions and sizes. In this study, we adopted YOLOv8 deep learning model as the framework for our experiment. YOLOv8 is an efficient and accurate real-time object detection network [28].

**Figure 2:**
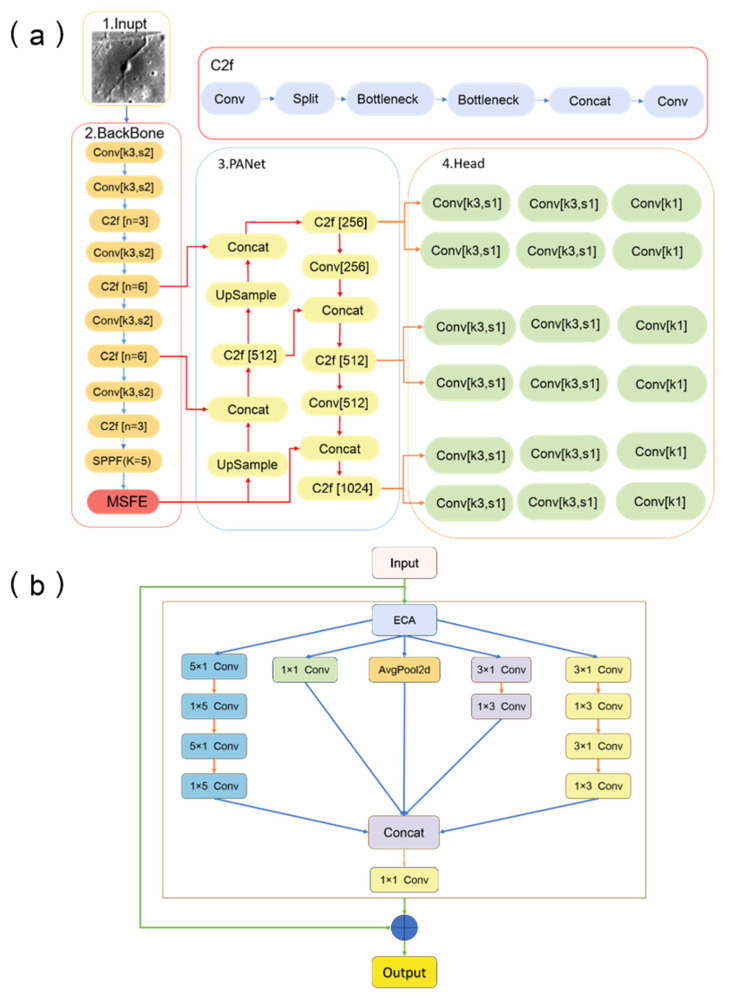
(**a**) DM-YOLO structure formed by the addition of the MSFE module to the YOLOv8 model. (**b**) Structure of the MSFE module.

The YOLOv8 network consists of four main modules: input, backbone network, neck network, and head network [29]. The input module is responsible for resizing and pre-processing the input image to meet the requirements of the subsequent network. The backbone network employs efficient convolution operations, such as lightweight convolution, depthwise-separable convolution, and adaptive convolution structures, to achieve efficient feature extraction and optimize computation speed. In addition, the backbone adopts the C2f feature aggregation module (Figure 2a), which is a lightweight residual-based structure designed to enhance gradient propagation and multi-scale feature reuse. The C2f block enables efficient feature interaction across layers while maintaining computational efficiency, making it suitable for extracting subtle morphological variations in lunar DMD imagery. Specifically, the backbone network in YOLOv8 uses network layers with adaptive learning capabilities, which can automatically adjust network weights based on the features of the input image, thereby improving the ability of the network to recognize objects of various sizes. The neck network uses an improved feature fusion module, which enhances the fusion of features at different scales by incorporating feature pyramid networks (FPNs) and path aggregation networks (PANets), allowing this module to better preserve fine-grained spatial and semantic information, reduce information loss, and improve the accuracy of small-object detection. The head network provides precise localization and classification of the objects through an enhanced detection module. YOLOv8 employs a multi-layer convolution structure and feature regression strategies in the head network, enabling the network to handle object detection tasks with different categories, scales, and complexities. Furthermore, YOLOv8 introduces a more efficient target-box regression strategy, leveraging global context information to further improve detection accuracy. Overall, YOLOv8 performs excellently in terms of both speed and accuracy, with its innovative network structure providing enhanced robustness and higher detection performance in complex object detection tasks.

To improve the performance of YOLOv8 in detecting lunar DMDs, this study has improved the backbone network of YOLOv8. Specifically, the YOLOv8 model was enhanced by introducing a MSFE module with an attention mechanism into the backbone network, strengthening the ability of the model to detect targets at different scales. This integration is motivated by the pronounced scale variability and low-contrast characteristics of lunar DMDs, rather than by a general architectural modification objective. The MSFE module improves recognition accuracy for small objects and complex scenes by parallel processing and fusing multi-scale features [30]. Through multi-scale feature fusion, the module enables the network to preserve large-scale contextual information while enhancing fine-scale morphological details that are critical for DMD identification. The dual-branch configuration of the MSFE module is specifically designed to address the heterogeneous characteristics of lunar DMDs. Regional dark mantle deposits (RDMDs) often exhibit relatively homogeneous large-scale textures, whereas localized dark mantle deposits (LDMDs) show stronger local contrast and irregular boundaries. By processing feature maps at different receptive fields simultaneously, the MSFE structure allows one pathway to maintain global morphological continuity while 1.25 another enhances fine-scale local variations, improving detection robustness across different DMD scales. Compared with single-scale or purely channel-based attention strategies, the MSFE design emphasizes multi-branch feature fusion and receptive-field diversity, which is particularly beneficial for separating diffuse DMD boundaries from shadows and low-albedo basaltic terrains. Moreover, this module optimizes the feature extraction process while maintaining model accuracy, thus further enhancing the performance of YOLOv8 in various object detection tasks. In the context of this study, such optimization is particularly important for global-scale lunar surface analysis, where computational efficiency and robustness are required.

To provide more detail, the MSFE module consists of components such as a channel attention mechanism (ECA) along with average pooling and spatially separable convolution. The ECA mechanism adaptively recalibrates channel-wise feature responses by modeling local cross-channel interactions without dimensionality reduction, allowing the network to emphasize informative feature channels while suppressing less relevant ones with minimal computational overhead. This design significantly enhances the feature expression capability of the network while preserving efficiency. The channel attention mechanism is used to adaptively reweight feature responses, allowing the network to emphasize informative morphological patterns associated with DMDs while suppressing background responses caused by shadows or albedo variations. The improved YOLOv8 structure employed in this work is presented in Figure 2.

The MSFE module is flexibly configured with sub-modules based on task characteristics and model architecture differences, aiming to accurately capture multi-scale features in the image. As illustrated in Figure 2b, residual connections are introduced within the dual-branch design to preserve original feature information during multi-scale processing. These residual pathways help maintain large-scale morphological context while preventing information degradation caused by repeated convolution operations, which is particularly beneficial for detecting diffuse pyroclastic deposits under complex lunar terrain conditions. It consists of the following dual-branch structure. The first branch employs residual connections, a classic architecture optimization technique in deep learning that helps alleviate gradient vanishing through skip connections. With adaptive tuning and careful data flow control, it integrates deeply into the overall architecture and accelerates model training. The second branch integrates the ECA, average pooling, and spatial convolution layers, working synergistically to efficiently extract DMD feature information. Together, the two branches complement each other, enhancing the performance of the MSFE module.

The ECA focuses on enhancing feature selection capabilities without introducing excessive computational burden. ECA uses global average pooling (without dimensionality reduction) to aggregate features and then adaptively determines the kernel size k, after which it performs a 1D convolution on the feature map with kernel size k. The final channel attention vector is obtained using the sigmoid function [31]. The calculation process of the ECA is as follows:

(1) $$w=F_{ECA}\left(X\right)=\sigma (Conv1D(GAP(X)))$$

(2) Y=w

In Equation (1), σ represents the sigmoid function, and Conv1D refers to the 1D convolution with a kernel size of k, where k is adaptively determined based on the channel dimension C. GAP(X) represents the global average pooling (GAP) operation, which performs GAP on each channel of the input feature map and converts it into a channel-level statistical vector. W denotes the channel attention vector. Finally, X and Y represent the input feature map and the output feature map, respectively.

(3) $$K=\psi \left(C\right)={\left|\frac{\log_{2}\left(C\right)}{\gamma }+\frac{b}{\gamma }\right|}_{odd}$$

where |x|odd denotes the closest odd number to x, γ and b are hyperparameters, and ψ(C) represents a function mapping relationship that generates specific output values based on the input channel dimension c. In this experiment, values of 2 and 1 were assigned to γ and b, respectively.

Average pooling performs downsampling by calculating the average value of the pixels in the corresponding region of the input feature map on the basis of the predefined kernel size. Since it has no parameters to optimize, it prevents overfitting by not excessively conforming to the nuances of the training data. The calculation of average pooling is as follows:

(4) $$F=\frac{1}{s\times s}\sum_{i=1}^{s}\sum_{j=1}^{s}X_{ij}$$

In Equation (4), s represents the kernel size, and Xij denotes the pixel value at point (i,j) within the computation region.

Spatially separable convolution is a convolution operation in which a standard convolution is decomposed into two independent operations: one is convolution in the spatial domain, and the other is convolution across channels. This method is typically used to reduce computational complexity and improve processing efficiency. Spatially separable convolution splits a standard convolution operation into multiple smaller kernel operations along the spatial dimension.

(5) $$\left[\begin{array}{ccc} w_{11} & \cdots & w_{1k}\\ \vdots & \ddots & \vdots \\ w_{k1} & \cdots & w_{kk} \end{array}\right]=\left[\begin{array}{c} x_{1}\\ \vdots \\ x_{k} \end{array}\right]\times \left[\begin{array}{ccc} y_{1} & \cdots & y_{k} \end{array}\right]$$

(6) $$w_{ij}=x_{i}\times y_{j},(1\leq i,j\leq k)$$

In Equation (5), wij represents the value at point (i,j) in the k × k convolution, xi represents the value at point (i,1) in the k × 1 convolution, and yj represents the value at point (1,j) in the 1 × k convolution. The number of parameters for the k × 1 convolution and the 1 × k convolution is (k + 1) + (1 + k) = 2 k, which is smaller than the number of parameters for a k × k convolution when k > 2. Spatially separable convolution reduces the number of parameters in the module, accelerates model computation, and improves model accuracy.

In the MSFE module with an attention mechanism [32], the input features are first enhanced by the ECA module, which applies channel attention. This multi-branch convolution strategy allows the network to simultaneously capture directional large-scale patterns and localized texture variations, which correspond to the morphological diversity of RDMDs and LDMDs. Such a design reflects a task-oriented adaptation of the feature extraction process for lunar geological imagery rather than a generic architectural modification. The features are then divided into multiple branches for convolution processing at different scales. The first branch uses a series of 5 × 1 and 1 × 5 convolution kernels to extract large-scale features from different directions; the second branch employs a 1 × 1 convolution for channel compression; and the third and fourth branches use 3 × 1 and 1 × 3 convolutions to extract smaller-scale local information. Additionally, some of the features undergo downsampling through average pooling to provide rich contextual information. The output features from each branch are then concatenated and further compressed via a 1 × 1 convolution. Finally, the multi-scale features are fused with the initial input features and passed to the next module to enhance the ability of the model to detect objects of different sizes.

The improved processing flow of the YOLOv8 model enhances its efficiency and accuracy, particularly in object detection tasks, combining multi-scale feature extraction with rapid inference capabilities while still allowing it to maintain high detection precision. This model integrates significant features from other models, achieving superior functionality, speed, accuracy, and robustness in image detection, segmentation, and classification. As a result, DM-YOLO becomes an ideal choice for identifying lunar DMDs on the surface of the Moon.

The software environment used in this study includes the Windows operating system, Python 11.3, CUDA 11.3, and PyTorch 2.5.1. To improve model detection accuracy, the initial parameter values were set using a pre-trained model developed by Ultralytics, which was trained on the DMD dataset. The image size for training and testing was set to 640 × 640, with a batch size of 16, and 100 epochs were used. The momentum, initial learning rate, weight decay regularization, and other parameters were automatically optimized during YOLOv8 model training. The model was tested after the training process was completed.

### 2.4. Accuracy Evaluation

To objectively evaluate the performance of the model in detecting lunar DMDs, this study employs evaluation metrics such as precision (P), recall (R), and mean average precision (mAP) [33]. The P and R for DMD detection can be calculated using the following formulas:

(7) $$P=\frac{TP}{TP+FP}$$

(8) $$R=\frac{TP}{TP+FN}$$

In Equations (7) and (8), TP represents true positives and refers to the number of correctly detected targets, FP represents false positives and refers to the number of false alarms, and FN (false negative) represents false negatives and refers to the number of missed detections. In the DMD detection task, high precision means that the model has fewer false positives when identifying targets, whereas high recall means that the model can identify an increased number of actual targets. The balance between precision and recall is crucial for evaluating the overall performance of a model.

In object detection tasks, mAP is an important evaluation metric that measures the average precision across all categories. mAP@0.5 is the average precision computed for a threshold intersection of union (IoU) of 0.5. Specifically, a detection is considered successful only when the overlap (IoU) between the predicted bounding box and the ground truth box is greater than or equal to 0.5. In this case, the calculation of the AP is similar to that described above, with the IoU threshold fixed at 0.5. Thus, the formula for calculating the mAP is as follows:

(9) $$AP=\int_{0}^{1}P(R)$$

(10) $$mAP=\frac{1}{C}\sum_{i=1}^{C}AP_{i}$$

(11) $$mAP@0.5=\frac{1}{C}\sum_{i=1}^{C}AP@0.5_{i}$$

In Equations (9)–(11), AP represents the average precision. The AP value is the area under the curve formed by P and R between 0 and 1. mAP represents the mean average precision, where C denotes the number of target categories and i is the category index. mAP@0.5 refers to the mean average precision when the IoU threshold is set to 0.5.

## 3. Experimental Results

### 3.1. Model Training Results

In this experiment, a comparative study was conducted to evaluate the impact of the MSFE module on training the YOLOv8 model for detecting DMDs, with all performance metrics in Table 1 calculated on the validation dataset. To comprehensively assess the effectiveness of the proposed DM-YOLO framework, we conducted experiments not only with different attention configurations based on YOLOv8 but also with several representative object detection models, including RetinaNet, Faster R-CNN, YOLOv5, and YOLOv7. The performance metrics included precision (P), recall (R), and mean average precision at 50% IoU (mAP@0.5).

Table 1 presents the performance comparison under identical training settings. Traditional two-stage and anchor-based detectors, such as RetinaNet and Faster R-CNN, show relatively lower performance on the DMD dataset, indicating challenges in handling diffuse boundaries and low-contrast features in lunar imagery. YOLOv5 and YOLOv7 achieve improved performance compared with earlier models; however, their results remain lower than those of the YOLOv8-based variants, suggesting that improved feature representation plays an important role in this task.

Among the YOLOv8-based models, the original YOLOv8 achieved P, R, and mAP@0.5 values of 69.9%, 68.0%, and 69.8%, respectively. The ML-YOLOv8 model, which integrates the mixed local channel attention (MLCA) mechanism, slightly improved performance (70.8%, 67.8%, and 70.2%), demonstrating the effectiveness of channel-wise feature enhancement. The PN-YOLOv8 model, incorporating the ParNet attention module, further improved the ability to capture long-range dependencies, resulting in P, R, and mAP@0.5 values of 76.5%, 68.0%, and 71.2%, respectively.

To provide a representative comparison with alternative multi-scale attention strategies, an MSAA-based variant (MS-YOLOv8) was also evaluated. Although MS-YOLOv8 improves certain aspects of feature representation, its overall performance (68.7%, 61.5%, and 62.3%) remains notably lower than that of DM-YOLO, indicating that generic multi-scale attention alone may not be sufficient for addressing the extreme scale variation and background confusion characteristic of lunar DMD imagery.

Finally, the proposed DM-YOLO model, integrating the attention-based MSFE module, achieved the best performance (P = 83.9%, R = 83.2%, and mAP@0.5 = 84.2%). Compared with the original YOLOv8 model, DM-YOLO improved precision by 14.0%, recall by 15.2%, and mAP@0.5 by 14.4%. These results indicate that while channel attention (MLCA) and long-range interaction modules (ParNet) enhance feature representation, the MSFE module provides stronger multi-scale feature aggregation capability, which is particularly beneficial for distinguishing DMDs from shadows and low-albedo basaltic terrains under global-scale lunar conditions.

In conclusion, the improved DM-YOLO model demonstrates superior robustness and detection accuracy compared with both conventional detectors and alternative attention-based YOLO variants, highlighting the effectiveness of task-oriented multi-scale feature extraction for lunar DMD identification.

### 3.2. Elemental Validation of Predicted DMDs Using FeO and TiO_2_ Maps

In this experiment, the YOLOv8 model was modified to DM-YOLO and achieved the expected training results. The trained weight files were then used to predict potential lunar DMDs. During the global prediction stage, a confidence threshold of 0.25 was applied to filter low-confidence detections. By performing forward propagation on lunar image slice data and weight files, the model identified several potential DMDs. To validate the accuracy of the predicted potential lunar DMDs, initial manual verification was conducted to remove obvious shadow or image interference areas. The remaining potential lunar DMDs were then marked on the lunar topographic map.

**Figure 3:**
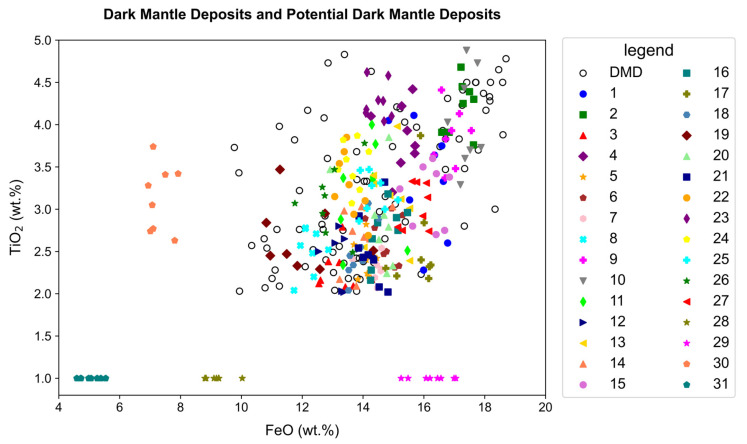
The unfilled circular points in the figure represent the FeO and TiO_2_ contents of the known lunar DMDs. The other points indicate the FeO and TiO_2_ contents of the potential lunar DMDs.

The experiment used the FeO abundance map derived from Clementine UVVIS (with a spatial resolution of approximately 200 m per pixel) to preliminarily validate the model-predicted DMDs, and used the TiO_2_ abundance map derived from the LROC WAC (with a spatial resolution of approximately 100 m per pixel) as an auxiliary geochemical reference to support the interpretation of compositional characteristics. Considering the spatial and radiometric resolution differences between the two datasets, we extracted FeO and TiO_2_ values by averaging over a 3 × 3 pixel window centered at each sampling point to minimize registration error and reduce pixel-level noise. Subsequently, potential lunar DMD regions were marked on the lunar topographic map, and random samples were taken from 10 known lunar DMD regions. In both the predicted and reference DMD regions, 8 random sampling points were selected for each region to analyze the percentage content of FeO and TiO_2_. These values were compared with the FeO and TiO_2_ data from known DMD regions to assess the accuracy of the predictions of the model. By plotting scatter diagrams for potential lunar DMDs and known DMDs, the accuracy of the potential DMDs was further validated. This study used the original DMD sampling data to validate the potential DMD sampling data, as shown in Figure 3.

This study uses scatter plots to compare the distributions of FeO and TiO_2_ contents between the model-predicted potential DMDs and known DMDs, allowing for the comparison of elemental data between known and potential DMDs to validate the identification performance of the model. The unfilled circular points in Figure 3 represent the distribution of known DMDs and reflect a specific distribution pattern in the FeO and TiO_2_ contents of these DMDs. Notably, these known DMDs are generally concentrated within certain ranges of FeO and TiO_2_ contents and form several clusters; however, since mare basalts may also exhibit overlapping compositions, these ranges are not uniquely diagnostic of DMDs. Therefore, the FeO abundance map was used to preliminarily validate the geochemical characteristics of the predicted DMDs, while the TiO_2_ abundance map served as an auxiliary reference to support compositional interpretation. These geochemical datasets were not treated as definitive classification criteria. Additionally, M^3^ spectral data were employed to further verify the mineralogical nature of the predicted deposits. The other colored points represent the FeO and TiO_2_ contents of the potential DMDs predicted by the model. These values were not predicted directly by the model but derived from existing datasets—specifically, the FeO abundance map from Clementine UVVIS and the TiO_2_ abundance map from LROC WAC—at the corresponding coordinates of the model-predicted regions. To improve reliability, data points that significantly deviated from the typical FeO ranges of known DMDs—particularly those with abnormally low TiO_2_ values (e.g., exactly 1%) but highly variable FeO—were excluded from the final analysis. These outliers were considered to result from potential limitations in TiO_2_ abundance estimation or local surface anomalies, and thus were not representative of actual DMDs. By observing the point distribution of the model-predicted DMDs, it can be seen in Figure 3 that the distribution areas of the FeO and TiO_2_ data points for some of the model-predicted DMDs highly overlap with those of known DMDs. This suggests that these potential DMDs have chemical compositions consistent with those of known DMDs, which grants the model greater authenticity and reliability.

Specifically, in Figure 3, the predicted DMDs show a consistent distribution trend of FeO and TiO_2_ content with that of known DMDs, validating the accuracy of the model identification results to some extent. However, Figure 3 also shows some discrepancies between the model-predicted DMDs and known DMDs. Some of these potential DMDs deviate from the typical ranges of FeO or TiO_2_ content, forming isolated points or exhibiting abnormal distributions.

These discrepancies may result from various factors, including uncertainties in the model prediction process, natural geochemical heterogeneity of the lunar surface, limitations in satellite image processing, or inaccuracies in elemental abundance estimation. In particular, the derivation of FeO and TiO_2_ values from remotely sensed data is subject to spatial resolution constraints and radiometric calibration uncertainties, especially for glass-rich or compositionally complex materials. Overall, the FeO and TiO_2_ abundance data serve as supportive geochemical references for evaluating potential DMD candidates rather than strict classification criteria. Since TiO_2_ abundance alone is not a definitive indicator of DMDs, further mineralogical verification was conducted using M^3^ hyperspectral data for each newly identified candidate, thereby ensuring the robustness and reliability of the final DMD confirmation.

### 3.3. Spectral Validation Using M^3^ Data

To further enhance the scientific credibility of the model’s predictions, we performed spectral validation of the identified potential dark mantle deposit (DMD) regions using hyperspectral data from the Moon Mineralogy Mapper (M^3^), in addition to FeO and TiO_2_ abundance verification. M^3^ provides spectral coverage from 460 to 3000 nm and accurately captures the reflectance characteristics of lunar surface materials. Notably, it detects key absorption features near 1 μm and 2 μm associated with iron-bearing minerals and pyroxenes, which are critical for distinguishing volcanic glass from ordinary basalts [6,7].

**Figure 4:**
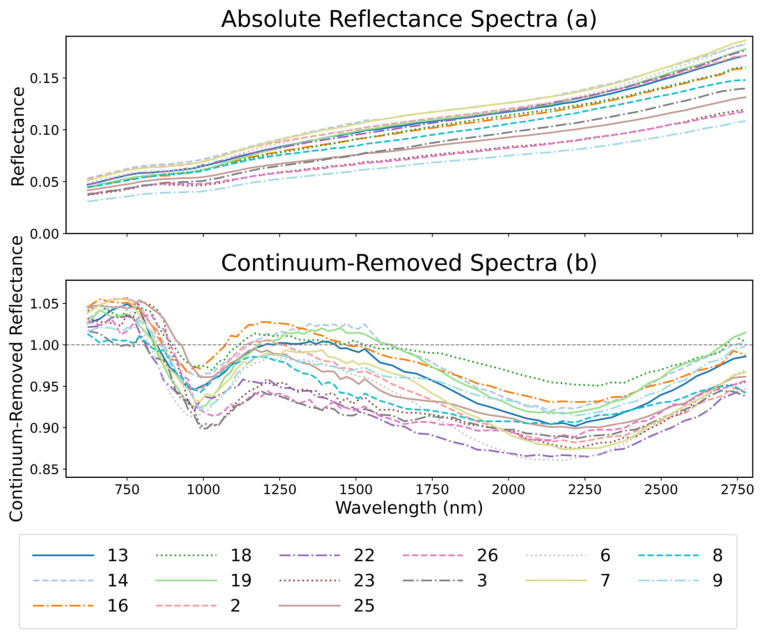

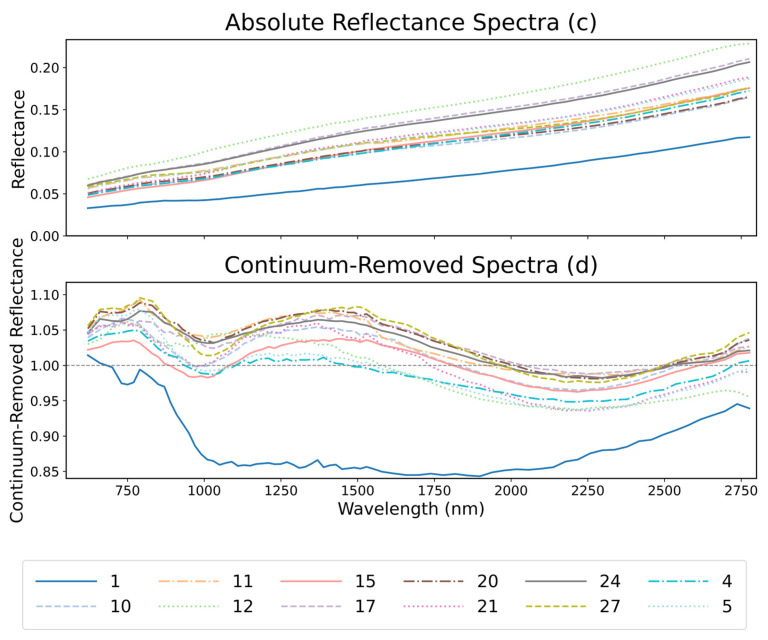
Spectral validation of model-predicted DMDs using M^3^ data. (**a**,**b**) Correctly predicted DMD region, showing low reflectance and well-defined absorptions near 1 μm and 2 μm; (**c**,**d**) misclassified region, exhibiting high reflectance and weak, asymmetric absorption features inconsistent with typical DMDs.

By comparing the original reflectance spectra with their continuum-removed counterparts, variations in mineral composition across the lunar surface can be effectively identified. This enables further discrimination between genuine pyroclastic deposits (i.e., DMDs) and other geologic units. Figure 4 illustrates two representative examples of spectral analysis based on model predictions: subfigures (a–b) correspond to a correctly identified DMD region, while (c–d) represent a misclassified area. In the reflectance spectra (Figure 4a,c), the correctly predicted DMD exhibits a generally low albedo with a smoothly increasing spectral trend toward longer wavelengths. This is consistent with the typical spectral behavior of DMDs, which are characterized by low reflectance and elevated Fe and Ti content. In contrast, the misclassified region displays a higher overall reflectance and lacks any clear absorption features, suggesting that its composition may not include volcanic glass or iron-rich pyroclastics. In addition to these general factors, several misclassified regions shown in Figure 4c,d exhibit irregular illumination geometry, fragmented terrain morphology, and mixed compositional signatures that resemble pyroclastic deposits in morphology but differ in geological origin. Such conditions increase intra-class variability and reduce boundary clarity, making it difficult for the model to distinguish between diffuse DMDs and visually similar background units.

In the continuum-removed spectra (Figure 4b,d), the correctly predicted region shows well-defined absorption features near 1000 nm and 2000 nm. These features exhibit considerable band depth, broad full width at half maximum (FWHM), and good symmetry—traits commonly associated with high-Fe glass or pyroxene-rich compositions. Such spectral characteristics are widely regarded as diagnostic of lunar DMDs.

In contrast, the misclassified region displays only weak and irregular fluctuations near the expected absorption positions. The band depths are shallow, the shapes are asymmetric, and the FWHM values are narrow, indicating a lack of the double absorption signature typically observed in pyroclastic deposits. These spectral traits suggest that the region may have been misidentified due to interference from local shadows, low-albedo basalts, crater rims, or surface maturity effects.

This spectral verification not only provides physical support for the accuracy of the model’s predictions but also reveals potential causes for misclassification. On one hand, correctly predicted samples exhibit spectral signatures consistent with known DMDs, confirming the model’s ability to capture relevant geologic phenomena. On the other hand, analysis of misclassified regions highlights the model’s vulnerability to confusion in areas with complex topography or ambiguous material composition.

The findings of this section demonstrate both the feasibility and necessity of integrating hyperspectral data for post-prediction validation. They also highlight the complementary strengths of deep learning models and traditional spectral analysis. In the challenging lunar environment, where lighting conditions and surface materials vary drastically, combining image-based detection, geochemical abundance assessment, and spectral verification provides a more robust and comprehensive framework for lunar resource exploration and site selection.

### 3.4. Global Distribution Map of DMDs

**Figure 5:**
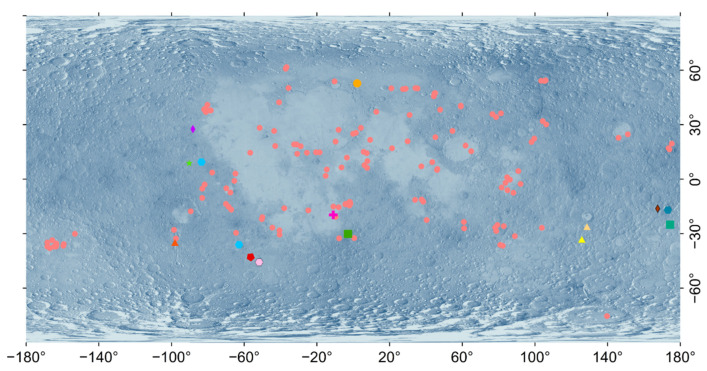
Spatial distribution of known and newly identified lunar DMDs. The pink circles represent the known DMDs, while the other colored symbols represent the newly predicted DMDs, categorized by different verification results based on M^3^ data analysis.

After the prediction results were validated, any anomalous potential lunar DMDs identified on the marked lunar topographic map were removed, leaving only the points that matched the chemical composition of the known lunar DMDs. In previous studies, more than 100 lunar DMDs have been identified globally [34]. In this experiment, the improved DM-YOLO model was used to predict DMD locations over the entire lunar surface, and after the prediction results were validated, 15 new lunar DMDs were identified. The known lunar DMDs and the predicted DMDs are presented in Figure 5.

As shown in Figure 5, the potential DMDs marked on the lunar topographic map (other colored symbols) exhibit notable spatial proximity to many of the known DMDs (pink circles), especially within geologically active regions such as low and mid-latitudes. This spatial relationship suggests that the model effectively identifies regions with similar geological or geomorphological characteristics conducive to DMD formation, aligning with existing distributions. This result indicated that after developing the DM-YOLO model, the predicted deposits generally reflected the distribution pattern of lunar DMDs. As shown in Figure 5, the known distribution of lunar DMDs is concentrated in the low- and mid-latitude regions of the Moon. Most of the newly discovered potential deposits were located close to known deposits, but additional distribution points emerged in certain areas, such as those between 130° and 180° east longitude and 10° and 35° south latitude. These new points may represent potential DMDs that were previously overlooked. The newly predicted deposits were mostly near lunar maria or impact crater regions, which corresponded to the typical formation environment for DMDs. In conclusion, these newly identified potential deposits provide an important supplement to the study of lunar DMDs, though further geological and mineralogical validation is necessary to confirm their authenticity and scientific value. To support future research and mission planning, we have compiled a dataset that includes the spectral validation results of several newly identified DMD regions. This dataset integrates geographic coordinates, M^3^ image identifiers, and extracted spectral profiles, and is publicly available in the and is publicly available in Table S1 from the Supporting Information. The establishment of this DMD database lays a solid foundation for future deep learning–assisted planetary exploration and geologic mapping efforts.

## 4. Discussion

### 4.1. DMD Distribution Characteristics

**Figure 6:**
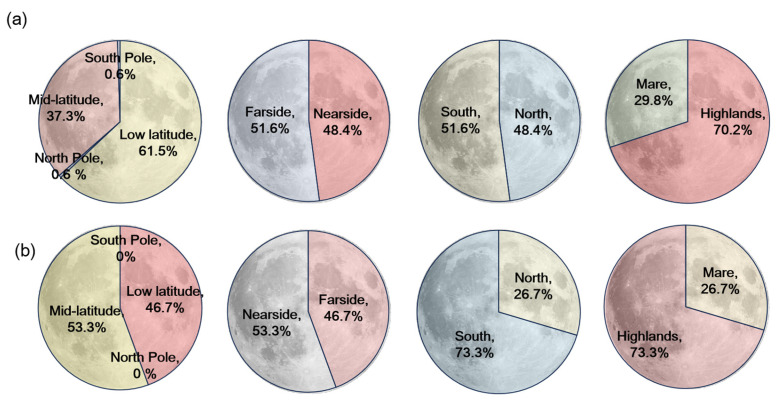
Global distribution characteristics of known and newly identified dark mantle deposits (DMDs), categorized by latitude, longitude (nearside/farside), hemisphere (north/south), and terrain (highlands/maria). Definitions: low-latitude = 0–30°, mid-latitude = 30–60°, polar = 60–90°. (**a**) Combined distribution of known and newly identified DMDs; (**b**) Distribution of newly identified DMDs.

Lunar dark mantle deposits (DMDs) exhibit a distinctive global distribution that reflects the Moon’s volcanic and geologic evolution. As shown in Figure 6, the majority of known and newly identified DMDs (61.5%) are located in the low-latitude region (0–30°), consistent with earlier observations that pyroclastic volcanism was more frequent and widespread in equatorial zones. This pattern likely results from a combination of factors, including thinner crust, enhanced thermal gradients, and abundant volcanic centers and impact basins in these regions. The mid-latitude region (30–60°) hosts 37.3% of these DMDs, indicating that this zone also played a significant role in early pyroclastic activity. In contrast, only 0.6% are found in the polar regions (60–90°), likely due to the presence of a thicker crust and impact modification processes that inhibited volcanic eruptions or obscured pyroclastic deposits. In terms of longitudinal distribution, the combined dataset of known and newly identified DMDs is relatively evenly split between the nearside (48.4%) and farside (51.6%) of the Moon. This indicates that pyroclastic volcanism was not limited to the more observable nearside but was also prominent on the farside, reinforcing the importance of farside volcanic processes that have often been overlooked due to limited observation coverage. Regarding terrain type, 70.2% of the DMDs are located in highland regions, while only 29.8% are distributed within mare basins. The predominance in highland areas may be attributed to better preservation of pyroclastic deposits, as these regions experienced less extensive basaltic flooding. In contrast, widespread mare volcanism may have obscured or buried early pyroclastic materials, making DMDs in those regions more difficult to detect using conventional remote sensing methods.

### 4.2. Distribution Characteristics of Newly Added DMDs

The spatial distribution of newly identified DMDs reveals both similarities and notable differences relative to known DMDs. As illustrated in Figure 6, 46.7% of newly identified DMDs occur in the low-latitude region, maintaining alignment with prior patterns. However, a higher proportion (53.3%) are now observed in the mid-latitude region, indicating that the improved method is capable of detecting less prominent or previously unrecognized deposits in these areas. No new DMDs were identified in the polar regions, in line with their inhospitable conditions for volcanic activity and unfavorable lighting geometry for remote detection. From a hemispheric perspective, 73.3% of the new DMDs are distributed in the southern hemisphere, which contrasts with the more balanced north–south split among known DMDs. This shift may reflect the algorithm’s enhanced ability to identify deposits in geologically complex terrains with historically limited coverage. In addition to algorithmic factors, geological and observational conditions may also contribute to this distribution pattern. Many newly identified candidates occur in the southern highlands, including regions influenced by the South Pole–Aitken Basin, where rugged terrain, heterogeneous surface composition, and complex illumination conditions can hinder traditional visual interpretation of diffuse pyroclastic deposits. The use of the illumination-normalized LROC WAC Global Morphology Map may reduce lighting-related biases and allow subtle morphological signatures to be more effectively captured by the deep learning model. The longitudinal distribution of newly identified DMDs also shows a slight shift: 53.3% are on the nearside, and 46.7% on the farside. This divergence from the known DMD distribution may indicate better detection performance in the high-albedo and morphologically varied nearside highlands. In terms of terrain classification, 73.3% of the newly identified DMDs are located in highlands, while only 26.7% are found in maria, reinforcing the method’s improved sensitivity to pyroclastic deposits in rugged or compositionally diverse terrains. The newly identified DMD candidates display diverse morphological expressions, ranging from diffuse sheet-like deposits distributed across highland terrains to smaller localized patches associated with irregular depressions or fissure-related structures. This variability suggests that some deposits may have been overlooked in previous visual surveys due to weak contrast and complex geological backgrounds.

The improved DMD identification method significantly enhances the ability to capture early volcanic activity deposits, especially in complex geological environments. By integrating multi-source data and optimizing deep learning models, the improved method reveals more volcanic debris, particularly in areas that are difficult to identify with traditional methods. This observed hemispheric difference should therefore be interpreted as the combined effect of geological setting, imaging characteristics, and model sensitivity to multi-scale morphological features, rather than as a purely algorithm-driven phenomenon. This not only improves our comprehensive understanding of lunar volcanic surface deposits but also provides more precise evidence for studying the spatiotemporal evolution, causal mechanisms, and geological development of lunar volcanic activity. Overall, the improved DMD identification and distribution characteristics indicate that this method has greater geological adaptability, enabling the identification of a broader range of volcanic deposits, especially in regions with complex geological environments, and offering new perspectives and research directions for a deeper understanding of the history of lunar volcanic activity, the evolution of the internal structure of the Moon, and geological changes on the lunar surface.

## 5. Conclusions

This paper proposed a lunar DMD identification method based on the DM-YOLO network. By integrating the MSFE module with the YOLOv8 model, this method significantly enhanced the accuracy and robustness of DMD detection. First, a DMD dataset was created using data from relevant studies, and the network architecture was adjusted to optimize feature extraction capabilities. To improve the performance of the model under complex terrain conditions, a new attention mechanism was introduced into the network, and various data-augmentation techniques were applied. The experimental results showed that the improved model achieved P, R, and mAP@0.5 values of 83.9%, 83.2%, and 84.2%, respectively, outperforming the original YOLOv8 model by 15.2%, 14.4%, and 14.0%, respectively. Compared with the existing YOLOv8 models, the proposed model offers significant advantages in both accuracy and efficiency. Furthermore, comparison of the DM-YOLO prediction results with FeO and TiO_2_ content data revealed a high degree of chemical consistency with known DMDs. This geochemical validation, combined with targeted M^3^ spectral analysis, further confirms the accuracy and reliability of the model in identifying lunar dark mantle deposits.

A comprehensive analysis of the global distribution of lunar DMDs revealed that DMDs are concentrated mainly in the low and mid-latitude regions of the Moon, with the low-latitude region showing the highest concentration. This distribution reflects the centralization and significance of volcanic activity. The proportion of DMDs found in the highlands is much greater than that in the maria regions, reflecting geological activities during the formation of the highlands, such as volcanic eruptions and impact events that created favorable conditions for the formation and deposition of DMDs. Additionally, the significant improvement in the detection capability of the new model resulted in the identification of weakly featured deposits on the far side and in complex geological environments, refining the global distribution data for DMDs. By supplementing and analyzing newly identified DMDs, this study deepened the understanding of the temporal and spatial evolution of lunar volcanic activity, providing new insights into lunar geological history and its internal evolution. These findings lay a crucial foundation for future lunar resource development and scientific exploration and offer key support for further research into the lunar evolution process.

This study demonstrated the potential of deep learning in lunar resource exploration by providing a novel solution, especially for tasks involving small-scale datasets such as DMD identification. In the future, this model can be further optimized and extended to planetary surface feature recognition tasks on other celestial bodies, such as Mars, Venus, and Mercury. By increasing the diversity of training datasets and adopting more advanced training strategies, the accuracy and generalizability of the model across different planetary environments can be significantly enhanced. As lunar exploration technology continues to advance, higher-resolution remote sensing imagery and more sample data could be used to optimize model performance and improve its adaptability and accuracy in real-world geological environments. Additionally, as algorithms evolve, the interpretability and visualization capabilities of the model should be further enhanced to provide geologists with deeper analysis and insights. Finally, this study offered theoretical support and a technical foundation for lunar resource exploration and deep-space exploration missions, with significant practical implications.

## Figures and Tables

**Table 1:** Performance comparison between YOLOv8 variants for DMD detection.

Model	P (%)	R (%)	mAP@0.5
Retina Net	52.2%	46.9%	41.7%
Faster R-CNN	22.2%	20.0%	16.83%
YOLOv5	62.1%	60.5%	61.3%
YOLOv7	59.1%	57.4%	58.0%
YOLOv8	69.9%	68.0%	69.8%
MS-YOLOv8	68.7%	61.5%	62.3%
ML-YOLOv8	70.8%	67.8%	70.2%
PN-YOLOv8	76.5%	68%	71.2%
DM-YOLO	83.9%	83.2%	84.2%

## Data Availability

The WAC and TiO_2_ abundance map data were provided by the LROC team https://data.lroc.im-ldi.com/lroc/view_rdr/WAC_TIO_2_ (accessed on 15 April 2025)). The FeO abundance map data were generated by the Clementine UVVIS team and are available via the NASA Moon Trek portal https://trek.nasa.gov/moon/ (accessed on 15 April 2025)). The M^3^ (Moon Mineralogy Mapper) spectral data used for further mineralogical validation were obtained from the NASA Planetary Data System (PDS) at https://pds-imaging.jpl.nasa.gov/volumes/m3.html (accessed on 15 April 2025)). The YOLOv8 object detection framework used in this study is based on the open-source implementation developed by Ultralytics and is publicly available at https://github.com/ultralytics/ultralytics (accessed on 15 April 2025)).

## Supplementary Materials

The following supporting information can be downloaded at: https://www.mdpi.com/article/10.3390/s26041318/s1, Table S1: Details of the dataset used for spectral validation of potential DMD regions.

## Abbreviations

The following abbreviations are used in this manuscript:

DMDs	Dark Mantle Deposits
RDMDs	“regional” dark mantle deposits
LDMDs	“localized” dark mantle deposits
LRO	Lunar Reconnaissance Orbiter
LROC	Lunar Reconnaissance Orbiter Camera
WAC	Wide Angle Camera
M^3^	Moon Mineralogy Mapper
MSFE	Multi-Scale Feature Extraction
