# Supplementary material for: Global Identification of Lunar Dark Mantle Deposits
Source: Sensors (Basel). 2026 Feb 18;26(4):1318. doi: 10.3390/s26041318 (PMC12944537; doi:10.3390/s26041318)
Supplement: Supplementary file 1 [file sensors-26-01318-s001.zip › sensors-4130483-supplementary.pdf]

Supporting Information for

Global Identification of Lunar Dark Mantle Deposits

Xiaoyang Liu[1], Jianhui Wang[1], Denggao Qiu[2,*], Jianguo Yan[2,3,*], Jean-Pierre Barriot[2,4], Yang Luo[1]

[1]School of Environmental and Disaster Management, University of Emergency Management, Sanhe, Hebei 065201, China.

[2]State Key Laboratory of Information Engineering in Surveying, Mapping and Remote Sensing, Wuhan University, Wuhan 430079, China.

[3]Xinjiang Astronomical Observatory, Chinese Academy of Sciences, Urumqi 830011, China

[4]Geodesy Observatory of Tahiti, University of French Polynesia, 98702 Fa'aa, Tahiti

*Corresponding author: Denggao Qiu (denggaoqiu@whu.edu.cn), Jianguo Yan (jgyan@whu.edu.cn)

Contents of this file

Table S1

Introduction

This supplementary material includes:
Table S1. Details of the dataset used for spectral validation of potential DMD regions

Table S1. Details of the dataset used for spectral validation of potential DMD regions

To evaluate the reliability of the predicted dark mantle deposit (DMD) regions, we compiled a dataset consisting of 15 representative sites that were confirmed through spectral validation using hyperspectral images from the Moon Mineralogy Mapper (M^3) onboard Chandrayaan-1. These sites span a wide range of lunar locations and include both absolute reflectance spectra and continuum-removed spectra derived from calibrated M^3 data. The geographic coordinates, M^3 image IDs, and extracted spectral profiles for each site are provided in the Supporting Information (Table S1).

ID	Longitude& Latitude	Image Used	Image Location	Absolute reflectance spectra	Continuum-removed spectra
2	2° 48′ 27″ W<br>30° 11′ 3″ S	M3G20090205<br>T211213_V01_<br>RFL	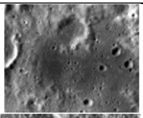	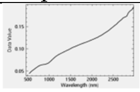	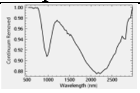
3	98° 3′ 6″ W<br>34° 52′ 52″ S	M3G20090213<br>T022112_V01_<br>RFL	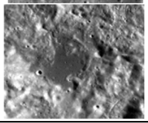	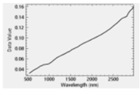	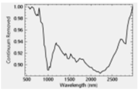

6	56° 19′ 58″ W<br>42° 32′ 43″ S	M3G20090612<br>T183813_V01_<br>RFL	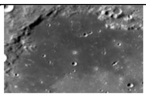	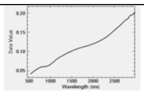	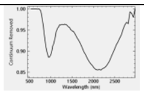
7	52° 8′ 41″ W<br>45° 46′ 25″ S	M3G20090612<br>T183813_V01_<br>RFL	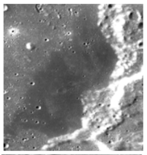	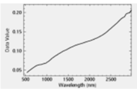	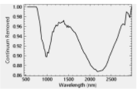
8	62° 46′ 37″ W<br>36° 4′ 20″ S	M3G20090419<br>T184909_V01_<br>RFL	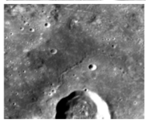	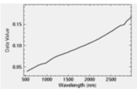	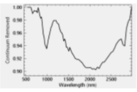
9	10° 59′ 50″ W<br>19° 34′ 10″ S	M3G20090206<br>T105850_V01_<br>RFL	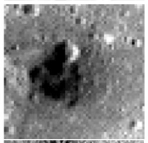	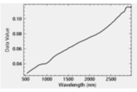	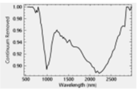
12	119° 6′ 40″ E<br>10° 25′ 9″ S	M3G20090530<br>T160828_V01_<br>RFL	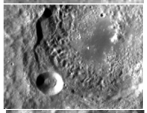	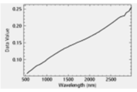	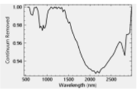
14	127° 48′ 28″ E<br>25° 55′ 34″ S	M3G20090529<br>T230608_V01_<br>RFL	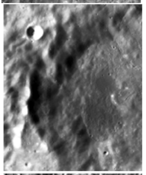	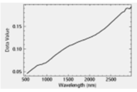	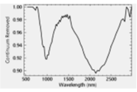
16	174° 22′ 3″ E<br>25° 4′ 29″ S	M3G20090720<br>T043741_V01_<br>RFL	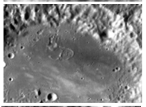	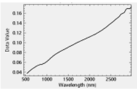	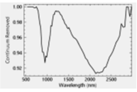
18	173° 43′ 2″ E<br>17° 1′ 16″ S	M3G20090720<br>T043741_V01_<br>RFL	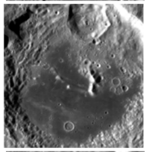	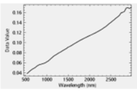	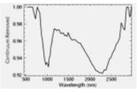
19	167° 36′ 20″ E<br>16° 13′ 39″ S	M3G20090623<br>T052831_V01_<br>RFL	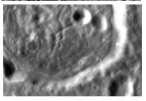	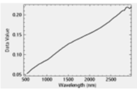	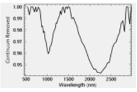
22	2° 12′ 46″ E<br>52° 43′ 59″ N	M3G20090608<br>T083142_V01_<br>RFL	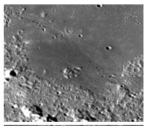	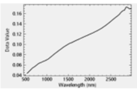	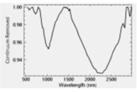
23	88° 8′ 5″ W<br>27° 33′ 48″ N	M3G20090212<br>T082712_V01_<br>RFL	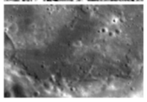	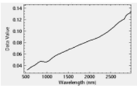	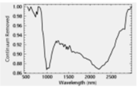
25	83° 28′ 44″ W<br>9° 25′ 35″ N	M3G20090212<br>T003453_V01_<br>RFL	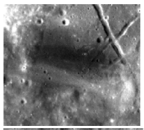	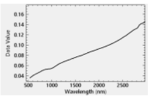	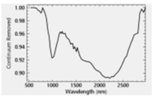
26	90° 15′ 3″ W<br>8° 48′ 4″ N	M3G20090212<br>T122313_V01_<br>RFL.IMG	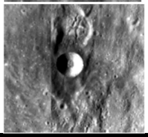	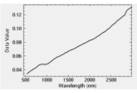	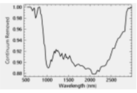

Note: The ID numbers correspond to those used in the preliminary FeO-based verification process and are retained for consistency.
